# The Association of Junk Food Consumption with Preadolescents’ Environmental Influences: A School-Based Epidemiological Study in Greece

**DOI:** 10.3390/children9121891

**Published:** 2022-12-02

**Authors:** Ioannis Gketsios, Thomas Tsiampalis, Alexandra Foscolou, Tonia Vassilakou, Aikaterini Kanellopoulou, Venetia Notara, George Antonogeorgos, Andrea Paola Rojas-Gil, Ekaterina N. Kornilaki, Areti Lagiou, Demosthenes B. Panagiotakos, Rena I. Kosti

**Affiliations:** 1 Department of Nutrition and Dietetics, School of Physical Education, Sports and Dietetics, University of Thessaly, 42132 Trikala, Greece; 2 Department of Nutrition & Dietetics, School of Health Science & Education, Harokopio University, 17671 Athens, Greece; 3 Department of Public Health Policy, School of Public Health, University of West Attica, 12243 Athens, Greece; 4 Laboratory of Hygiene and Epidemiology, Department of Public and Community Health, School of Public Health, University of West Attica, 12243 Athens, Greece; 5 Department of Nursing, Faculty of Health Sciences, University of Peloponnese, 22100 Tripoli, Greece; 6 Department of Preschool Education, School of Education, University of Crete, 74100 Rethymnon, Greece; 7 Faculty of Health, University of Canberra, Canberra 2617, Australia

**Keywords:** junk food, children, preadolescents, influences, parents, school, advertisements

## Abstract

The aim of the present study was to evaluate the impact of environmental influences on Greek preadolescents’ junk food consumption. A cross-sectional study, was conducted among 1718 preadolescents (mean (standard deviation(SD)) age: 11.2(0.8) years old; 54% girls) and their parents, during the school years 2014–2016. Parental and child characteristics were collected anonymously, through self-administered and validated questionnaires. Among others, junk food consumption was recorded, classifying children as low, moderate, and high consumers. The majority of the preadolescents were classified as at least moderate junk food consumers, while almost 3/10 children were classified as high junk food consumers. A significantly lower junk food consumption was observed among preadolescents with a healthier family environment, consisting of normal-weight parents who consume junk foods less frequently, prefer home-cooked meals and adhere more to the Mediterranean diet, while more frequent family meals were also associated with lower junk food consumption. In addition, influence from teachers and participation in extracurricular sports activities were significantly associated with lower junk food consumption, while advertisements were found to have a significant negative impact on preadolescents’ eating habits. Notwithstanding, peers were not found to influence their dietary choices in terms of junk food consumption. Both parents and teachers seem to be positive influencers on preadolescents’ low junk food consumption. The detrimental role of advertisements on junk food consumption is reconfirmed, while peers’ influence is not significant on junk food consumption. The need for urgent public health initiatives for the promotion of healthy dietary habits among preadolescents is warranted.

## 1. Introduction

According to the World Health Organization (WHO), almost one out of five children and adolescents aged 5–19 years old are overweight and obese, accounting for over 340 million “victims” of the obesity pandemic globally, while in Europe this figure is expected to rise by 1.3 million (each year) during the next few years [1]. The negative impact and the adverse health effects of childhood overweight and obesity have been thoroughly investigated by several research teams, showing that obesity is not only related to decreased social and psychological functioning of children [2], but also to the development of non-communicable diseases in adulthood [3,4]. The etiology of childhood obesity is multi-factorial, yet one of the most crucial factors contributing to its rise is poor eating habits, such as the inadequate intake of fruits and vegetables and the excessive eating of high-calorie unhealthy snacks and junk foods [5,6]. 

A recently conducted meta-analysis showed that children’s eating habits have grown increasingly far from a healthy and balanced diet [7], with almost 20% of children’s calorie intake coming from junk foods [7,8]. Both eating habits and food preferences are established during early childhood; however, food preferences change throughout life, and are influenced and driven by several biological, social, and environmental factors [9]. The modern way of life and society, as well as the long working hours of parents, often lead children to poorer eating habits and higher junk food consumption, yet literature also suggests that nutrition education in school settings, could help children adopt healthier consumption habits [10,11]. However, even in cases of healthy family and school environments, the “digital explosion” and the increased digitalization of unhealthy food environments [12], have a great negative impact on children’s eating behaviors and choices [13]. At the same time, during early adolescence, when parental influence is lessened [14], peers constitute another potential social determinant of preadolescents’ food preferences, albeit a recently conducted systematic literature review has demonstrated controversial findings in children and adolescents [15], supporting that peers’ influence may be both positive as well as negative on children and adolescents’ dietary habits. 

Thus, it is of utmost importance to identify both the social as well as environmental factors influencing children’s food choices [16] and in particular during preadolescence, which is the prelude of a metamorphosis that occurs in adolescence and is determined by physical, emotional, cognitive, and sociocultural changes [17] characterizing the transition from childhood to adolescence. In this respect, the critical neurobiological period of preadolescence is significant when behaviors and habits with long-term health implications are being shaped. Therefore, the aim of the present school-based, cross-sectional study was to evaluate the impact of environmental influences on Greek preadolescents’ junk food consumption. 

## 2. Materials and Methods

### 2.1. Design

This is a cross-sectional, school-based, observational study.

### 2.2. Setting

The study took place in the Athens metropolitan area, in the Heraklion city area (the capital city of the island of Crete), and in three cities of Peloponnese (Sparta, Kalamata, and Pyrgos) in southern Greece. The regions represent large urban and rural municipalities from southern Greece. The enrolment procedure was carried out during the school years 2014–2015 and 2015–2016. The schools participating in the present study were randomly selected from a list provided by the Greek Ministry of Education, and all students aged 10–12 were asked to participate. In total, 47 primary schools (32 in Athens metropolitan area, 5 in Heraklion, 3 in Pyrgos, 2 in Kalamata, and 5 in Sparta) were included. More information can be found elsewhere [18].

### 2.3. Sample

In total, 1728 students (795 boys) aged 10–12 years old, were enrolled in the study. All children studying at the 5th and 6th grades of primary school were eligible to participate; no exclusion criteria were applied. The participation rate of children ranged from 95% to 100% in all participating schools and areas. After checking the questionnaires’ completeness, the final working sample for the present analysis was n = 1718 preadolescents (mean (standard deviation (SD)) age: 11.2 (0.8) years old; 54% girls), while their parents were also invited to participate, with a 68.9% response rate being achieved (n = 1190; mean (SD) age- fathers: 45.8 (5.2) years and mothers: 41.5 (4.4) years). 

### 2.4. Bioethics

Before starting the study, approval was requested from the appropriate department of the Ministry of Education and Religious Affairs (code of approval F15/396/72005/C1 by the Institute of Educational Policy (1989) and was carried out following the principles of the Declaration of Helsinki. The investigators informed all people who were involved about the aims and procedures of the research. The students participated in the study after the written consent of their parents had been obtained.

### 2.5. Measurements

Specially trained health scientists/investigators (i.e., dietitians, registered nurses, physicians) conducted a face-to-face interview with each child and their parents, which lasted 10–15 min. Children were interviewed separately from their parents. 

#### 2.5.1. Children’s Characteristics

Each child completed a questionnaire specially developed for the study. In order to avoid errors and discrepancies, the investigators assisted children by giving practical examples when it was necessary. Each child was provided with a personal code by the school principal, in order for the questionnaires to be cross-referenced to those of their parents. The questionnaire retrieved information, among others, about basic socio-demographic (age, sex) and anthropometric characteristics (weight, height), as well as several other characteristics, including physical activity status, nutritional habits, as well as level of adherence to the Mediterranean diet. 

#### 2.5.2. Children’s Physical Activity Status

Children’s physical activity status, defined as their involvement in out-of-school activities such as participation in sports clubs, playing with others, jogging, and swimming, on a daily or weekly basis, was estimated through the standardized, validated, and reliable Physical Activity and Lifestyle Questionnaire (PALQ) [19]. 

#### 2.5.3. Children’s Anthropometric Characteristics

Specially trained investigators measured the necessary anthropometric measurements of children (height (in m), weight (in kg)) using a tape measure and a scale (with skin-tight clothing, to minimize measurement errors), so as to calculate their body mass index (BMI). Children’s weight status was evaluated through the age- and the sex-specific International Obesity Task Force (IOTF) body mass index cut-off criteria [20], and afterwards, they were categorized as underweight, normal-weight, overweight, and obese. Due to the limited number of children who were underweight and obese, under- and normal-weight categories, as well as overweight and obese categories, were combined.

#### 2.5.4. Children’s Nutritional Habits

Level of adherence to the Mediterranean Diet was evaluated through a special Mediterranean Diet quality index (i.e., KIDMED) [21] (score range −4 to 12; ≤3, very-low-quality diet; 4–7, need to improve the food pattern to adjust it to the Mediterranean one; ≥8, optimal Mediterranean diet), while a validated semi-quantitative Food Frequency Questionnaire (FFQ) was also used, in order to evaluate their dietary habits including all foods and beverages consumed by the general child population, and their dietary behaviors (i.e., breakfast consumption, number of meals per day, frequency of having meals with their parents, etc.) [22]. 

#### 2.5.5. Environmental Influencers on Children’s Behavior

The questionnaire also included statements regarding the environmental influencers on children’s habits. More specifically, children were asked who influenced their lifestyle and choices most (i.e., parents/relatives, advertisements, teachers, classmates).

#### 2.5.6. Parents’ Characteristics

Several parental sociodemographic characteristics (age), anthropometric characteristics [weight (kg) and height (m)], educational level (i.e., primary, secondary, university) and financial characteristics (income status under or over 18.000 €/year) were recorded by the children’s parents. 

#### 2.5.7. Parents’ Nutritional and Lifestyle Habits

Parents’ level of adherence to the Mediterranean diet was evaluated through a specially designed index, the MedDietScore (range 0–55), which assesses the level of adherence to the Mediterranean dietary pattern [23]. Higher values on this index indicate greater adherence to this pattern and, consequently, healthier dietary habits. Parents whose score was ≤25 units were classified as being “Away” from the Mediterranean diet, while parents whose score was >25 units were classified as being close/very close to the Mediterranean diet. In addition, a validated semi-quantitative food frequency questionnaire (FFQ) was also used, in order to evaluate their dietary habits, and their dietary behaviors (i.e., eating outside from home, ordering food from outside, etc.). Finally, parents’ lifestyle characteristics (smoking status (yes/no), physical activity status (not at all or at least 1–2 times per week)) were also recorded. 

#### 2.5.8. Parental and Children’s Junk Food Consumption

Following the methodology proposed by Boylan et al. (2017) [24], the junk food intake measure, both for the children, as well as for their parents, was based on the consumption of fried potatoes, soft drinks, chocolates/croissants/cookies, and potato crisps/salty snacks. Each food item was assigned a score of 0–5 depending on the frequency of food intake (0 being never/rarely and 5 being two or more times per day) so that the junk food intake measure ranged from 0 to 20 (0 being no junk food consumed). Afterwards, based on the tertiles of the junk food intake measure, both children and their parents were classified as: Low junk food consumers (score: 0–4), Moderate junk food consumers (score: 5–7), and High junk food consumers (score: 8–20). 

### 2.6. Statistical Analysis

Continuous variables are presented as mean values (standard deviation, SD) and categorical variables are presented as relative frequencies (%). Normality of the continuous variables’ distribution was tested through graphical (histograms, PP- plots, QQ- plots) and statistical means (Shapiro–Wilk test). The one-way analysis of variance (ANOVA) was used in order to investigate the association between the continuous characteristics and the frequency of junk food consumption (low, moderate, high), while the Pearson chi-square test was used in the case of the categorical characteristics. Multivariable multinomial logistic regression analysis was implemented in order to investigate the association between the preadolescents’ frequency of junk food consumption and the environmental (family, school, social) characteristics. Results are presented as odds ratios (OR) and 95% confidence intervals (CI) and compare the low junk food consumption group with the moderate and the high junk food consumption group. All results are adjusted for children’s age and sex, as well as for parents’ age, sex, BMI, physical activity, education level, income status and level of adherence to the Mediterranean diet. All statistical analyses were performed using SPSS software version 29.0 (SPSS, Inc. Armonk, NY, USA: IBM Corp) and the significance level was set at a = 0.05.

## 3. Results

### 3.1. Profile of High Junk Food Consuming Preadolescents

Table 1 presents the preadolescents and their parents’ characteristics, both in the total sample, as well as separately, according to the frequency of junk food consumption. Almost 6 out of 10 preadolescents (59.6%) were classified as at least moderate junk food consumers, while 27% of the total samples were classified as high junk food consumers. As depicted, boys (*p* < *0.001*), as well as preadolescents with a very low diet quality, were more likely to be high junk food consumers (*p* < *0.001*), while there was also an indication that overweight/obesity was more prevalent among high junk food consumers (*p* = *0.061* < *0.100*). As far as the parents’ characteristics are concerned, high junk food consuming preadolescents were more likely to have parents with both unhealthier smoking habits *(p* = *0.028)*, as well as unhealthier nutritional habits *(p* < *0.001*). Finally, it is also worth noting that the fathers of high junk food consuming preadolescents were characterized by lower educational level, when compared to moderate and low junk food consuming preadolescents *(p* = *0.057* < *0.100*). 

### 3.2. Family Environment and Preadolescents’ Frequency of Junk Food Consumption

After adjusting for several characteristics, as presented in Table 2, a healthier family environment was found to be significantly associated with lower junk food consumption from preadolescents. More specifically, preadolescents with low and moderate junk food consuming parents, were found to have 86% and 73% lower odds of being high junk food consumers, respectively, while at the same time, preadolescents with low junk food consuming parents had 43% lower odds of being moderate junk food consumers. Furthermore, a significantly lower consumption of junk food was also observed among preadolescents whose parents neither eat outside nor order their food from outside. Furthermore, higher parental adherence to the Mediterranean diet and better weight status, were also associated with significantly lower junk food consumption, as preadolescents whose parents were close/very close to the Mediterranean diet, as well as those whose parents were both normal-weight, had 70% and 51% lower odds of being high junk food consumers, respectively. Finally, preadolescents having meals with their parents on a more frequent basis (at least two times/week), were found to have almost two times higher odds of being low junk food consumers, instead of being high consumers. 

### 3.3. School, Social, Advertising Environment and Preadolescents’ Frequency of Junk Food Consumption

As depicted in Table 2, both the teachers, as well as the participation of preadolescents in extracurricular sports activities were found to have a significantly beneficial impact on their nutritional habits. In particular, preadolescents participating in sports activities have almost half the odds of being high junk food consumers when compared to those not participating in such activities, while at the same time preadolescents stating that their lifestyle and nutritional choices are being influenced mostly by their teachers, have 31% lower odds of being high junk food consumers. On the other hand, the TV environment and in particular advertising environment, was found to have a significant and negative impact on preadolescents’ nutrition, as those stating that their lifestyle and nutritional choices are being influenced mostly by advertisements, have 45% higher odds of being high junk food consumers. Notwithstanding, influence from classmates was found to have no association with preadolescents’ junk food consumption.

## 4. Discussion

To the best of our knowledge, the environmental determinants of preadolescents’ junk food consumption have been inadequately investigated. The present work revealed that almost 3 out of 10 preadolescents were high junk food consumers, with the boys, those having less healthy dietary behaviors and higher body weight, being more likely to consume junk foods on a more frequent basis. Moreover, preadolescents were favorably influenced, in terms of lower junk food consumption, by a healthier family and school environment through teachers, while they were unfavorably impacted by advertisements and media. As for their peers, they were not found to have any significant impact on this unhealthy dietary habit. Despite the potential limitations of the present study, such as the observational design, the public health messages are of paramount importance, underscoring the need for the early management of negative influences on preadolescent lifestyle habits, as long as school and family environment still play a critical role.

In line with other cross-sectional studies and the Centers for Disease Control and Prevention (CDC), Greek preadolescent boys were found to be more susceptible to higher junk food consumption [25], which in turn is strongly related to higher prevalence rates of obesity [26]. Within the family context, parents either having the responsibility for their children’s food choices or serving as role models, play a crucial role in shaping children’s dietary habits [16,27,28]. In line with previous literature, findings in the present work, show that a family environment with healthy habits may contribute to lower preadolescents’ junk food consumption, demonstrating the critical role that families play in promoting healthy behavior [27]. The findings of the present analysis are also in accordance with those of other studies suggesting that children are prone to adopt their parents’ dietary habits, as they have the biggest influence on them [11]. In a child’s ecological microenvironment, parents provide food and eating experiences such as family meals, as they represent an important opportunity for both controlling their children, as well as for interacting with them [29]. In this context, children model themselves on their parents’ eating and lifestyle habits [11] showing that the “visual” cues and experiences that preadolescents receive from their parents on a daily basis, greatly influence their eating habits. In addition, the present findings could be also attributed to the implementation of parental rules for moderate restriction, probably resulting in higher nutrition knowledge acquired by their children [30]. 

Moreover, in line with other studies, results showed that low junk food consumers are strongly influenced by their teachers, while they are also engaged in higher extracurricular sports activities. Schools are widely acknowledged as the ideal settings for the promotion of healthy behaviors in children [31], as teachers in school settings can further strengthen the nutrition knowledge acquired in a healthy family environment [32]. In accordance with our findings, previous research has also demonstrated the association between food and drink advertisements that children are exposed to, and their diet quality [33,34,35]. TV advertisements with figures of poorer eating habits and higher junk food consumption, may lead children and teenagers to adopt unhealthier nutritional habits before they are emotionally prepared to comprehend them and prior to making wise decisions [36].

Although there is still controversy in the literature [15], in agreement with other studies [37,38] findings from the present analysis showed that Greek preadolescents were not influenced by their classmates as regards their dietary habits. One could presume that many cognitive processes undergo alterations between childhood and adolescence [17] which are not completed during the pre-adolescent stage, resulting in the fact that preadolescents act more like children and accept adult influence to a greater extent. Based on the present results, it seems that Greek preadolescents tend to have a mindset towards child-like instead of adolescent-like, probably because they are still exposed to their parents’ and teachers’ influence in the traditional cultural environment.

### Strengths and Limitations

To the best of our knowledge, this is the first study evaluating the association of Greek preadolescents’ junk food consumption with environmental influences. However, the conclusions of the present study should be considered under the existing limitations. The cross-sectional design does not allow causal associations to be drawn. Another limitation could be the reporting bias due to the self-reporting questionnaires by schoolchildren. However, to reduce this type of bias and increase the validity of the given responses, trained investigators were present throughout the whole procedure of completing the questionnaire in schools to address any potential misconceptions.

## 5. Conclusions

Both parents and teachers seem to be positive influencers on low preadolescents’ junk food consumption. The detrimental role of advertisements on junk food consumption is reconfirmed while peers’ influence on preadolescents is not significant in junk food consumption. Future intervention studies should take into serious consideration both the family environment and teachers’ role in the promotion of healthy dietary habits from the public health perspective. Stakeholders should undertake their responsibility as regards the harmful effect of TV advertisements on children’s eating habits. A deeper understanding of how unhealthy or healthy behaviors co-occur within the family and school environments could inform the development of more effective prevention approaches. 

## Figures and Tables

**Table 1:** Preadolescents and their parents’ characteristics, both in the total sample, as well as separately according to the frequency of junk food consumption.

	Overall Sample (N = 1718)	Categories of Preadolescents’ Junk Food Consumption			*p-Value*
		Low (N = 694)	Moderate (N = 559)	High (N = 465)	
Children’s characteristics					
Age [years; Mean (SD)]	11.2 (0.8)	11.1 (0.8)	11.2 (0.8)	11.3 (0.8)	*0.054*
Sex [N (% Girls)]	558 (54.0)	415 (59.8)	301 (53.8)	212 (45.6)	*<0.001*
BMI [kg/m^2^; Mean (SD)]	19.2 (3.4)	19.3 (3.5)	19 (3.3)	19.5 (3.6)	*0.061*
Overweight/Obesity N (% Yes)	473 (27.5)	194 (27.9)	142 (25.4)	138 (29.6)	
KIDMED score [Mean (SD)]	5.0 (2.0)	5.0 (2.0)	5.0 (2.0)	4.0 (2.0)	*<0.001*
KIDMED score categories [N (% Very low diet quality)]	472 (32.5)	186 (26.8)	162 (29.0)	210 (45.2)	
Parents’ characteristics					
Father’s age [years; Mean (SD)]	45.8 (5.2)	45.6 (5)	46.1 (5.5)	45.8 (5.2)	*0.333*
Mother’s age [years; Mean (SD)]	41.5 (4.4)	41.5 (4.3)	41.6 (4.5)	41.4 (4.5)	*0.853*
Father’s educational level [N (% Higher education)]	685 (39.9)	298 (43.0)	226 (40.4)	160 (34.4)	*0.057*
Mother’s educational level [N (% Higher education)]	775 (45.1)	320 (46.1)	250 (44.7)	204 (43.9)	*0.822*
Income status [N (% >18,000 euros/year)]	868 (50.5)	355 (51.2)	283 (50.6)	230 (49.5)	*0.900*
Father’s BMI [kg/m^2^; Mean (SD)]	27.0 (3.7)	27.1 (3.9)	26.8 (3.3)	27.2 (4.0)	*0.429*
Mother’s BMI [kg/m^2^; Mean (SD)]	24.0 (4.0)	24.1 (4.2)	23.8 (3.8)	24.2 (3.8)	*0.375*
Parental obesity status [N (% At least one parent obese)]	1257 (73.2)	498 (71.8)	421 (75.3)	339 (72.9)	*0.535*
Parental smoking habits [N (% At least one parent smokes)]	955 (55.6)	366 (52.7)	302 (54.1)	290 (62.3)	*0.028*
Parental physical activity status [N (% None of the parents is physically active)]	409 (23.8)	153 (22.1)	148 (26.5)	107 (23.10	*0.314*
Parental level of adherence to the Mediterranean diet [N (% Away from the Mediterranean diet)]	861 (50.1)	285 (41.1)	248 (44.3)	324 (69.7)	*<0.001*

**Notes**: *p*-value was based on the one-way analysis of variance (ANOVA) in case of continuous characteristics and Pearson chi-square test in case of categorical characteristics. SD = standard deviation. BMI = body mass index. Parental level of adherence to the Mediterranean diet was estimated through the MedDietScore, and those scoring ≤ 25 units, were classified as being “Away” from the Mediterranean diet. Obesity status was defined as BMI > 29.9 kg/m^2^. KIDMED scores range from 4 to 12. Lower scores indicate low adherence to the Mediterranean diet while higher scores, high adherence to the Mediterranean diet (≤3, very-low-quality diet; 4–7, need to improve the food pattern to adjust it to the Mediterranean one; ≥8, optimal Mediterranean diet).

**Table 2:** Results of multinomial logistic regression analysis regarding the environmental effect on preadolescents’ likelihood of consuming junk foods.

			Categories of Preadolescents’ Junk Food Consumption					
			Moderate vs. Low			High vs. Low		
			OR	95% CI	*p*	OR	95% CI	*p*
Influence of family environment								
*Frequency of parental junk food consumption* *(Ref: High)*	*Low*		0.57	0.36–0.90	*0.016*	0.14	0.09–0.23	*<0.001*
	*Moderate*		0.93	0.58–1.48	*0.753*	0.27	0.17–0.45	*<0.001*
*Parents tend to eat outside* *(Ref: Yes)*	*No*		0.94	0.71–1.25	*0.660*	0.71	0.53–0.96	*0.026*
*Parents tend to order food from outside* *(Ref: Yes)*	*No*		0.69	0.52–0.90	*0.007*	0.55	0.41–0.74	*<0.001*
*Parental level of adherence to the Mediterranean diet* *(Ref: Away)*	*Close/Very close*		0.88	0.65–1.19	*0.408*	0.30	0.22–0.42	*<0.001*
*Parents’ obesity status* *(Ref: At least one parent obese)*	*Both parents normal- weight*		0.55	0.29–1.04	*0.067*	0.49	0.25–0.94	*0.033*
*Frequency of family meals* *(Ref: Less than 2 times/week)*	*At least 2 times/week*		0.86	0.63–1.19	*0.365*	0.58	0.43–0.79	*0.001*
Influence of school, social and advertising environment								
*Influence from teachers* *(Ref: No)*		*Yes*	0.83	0.65–1.08	*0.160*	0.69	0.52–0.92	*0.010*
*Influence from classmates* *(Ref: No)*		*Yes*	1.10	0.84–1.43	*0.490*	1.16	0.86–1.59	*0.330*
*Participation in sports activities outside school* *(Ref: No)*		*Yes*	0.83	0.62–1.12	*0.227*	0.55	0.41–0.74	*<0.001*
*Influence from Advertisements* *(Ref: No)*		*Yes*	1.15	0.63–1.21	*0.420*	1.45	1.02–2.08	*0.039*

**Notes**: Results are presented as odds ratios (OR) and 95% confidence intervals (CI). All models were adjusted for children’s age and sex, as well as for parents’ age, sex, BMI, physical activity, education level, income status and level of adherence to the Mediterranean diet.

## Data Availability

Data are available upon request.
